# Reducing the Environmental Impact of Dietary Choice: Perspectives from a Behavioural and Social Change Approach

**DOI:** 10.1155/2012/978672

**Published:** 2012-06-17

**Authors:** Andrew Joyce, Sarah Dixon, Jude Comfort, Jonathan Hallett

**Affiliations:** ^1^EACH Social and Community Health, 46 Warrandyte Road, Ringwood, VIC 3134, Australia; ^2^Department of Occupational Therapy, Monash University, Peninsula Campus, P.O. Box 527, Frankston, VIC 3199, Australia; ^3^Department of Health Promotion, School of Public Health, Curtin University, GPO Box U1987, Perth, WA 6845, Australia

## Abstract

Climate change is recognised as a significant public health issue that will impact on food security. One of the major contributors to global warming is the livestock industry, and, relative to plant-based agriculture, meat production has a much higher environmental impact in relation to freshwater use, amount of land required, and waste products generated. Promoting increased consumption of plant-based foods is a recommended strategy to reduce human impact on the environment and is also now recognised as a potential strategy to reduce the high rates of some chronic diseases such as cardiovascular disease and certain cancers. Currently there is a scant evidence base for policies and programs aiming to increase consumption of plant-based diets and little research on the necessary conditions for that change to occur and the processes involved in such a change. This paper reviews some of the environmental and health consequences of current dietary practices, reviews literature on the determinants of consuming a plant-based diet, and provides recommendations for further research in this area.

## 1. Introduction

Climate change is recognised as a significant public health issue and its impact on food security is one area of concern [1, 2]. Changes to climate also link with concerns in relation to land degradation, loss of biodiversity, and increases in input demands on the food system which all impact on food security [3]. These consequences on the food system will significantly create long-term impacts for the environment and public health, resulting in decreased food security [2]. There are a number of food and nutritional policies and strategies required to address these issues, and one domain requiring focus is shifting consumer behaviour [4]. World-wide there is an increase in demand for milk, meat, and eggs resulting from rising incomes, growing populations, urbanised populations, and preference choices [5, 6]. The sustainability of livestock is not as viable as that of crop production [7]; therefore, shifting consumer behaviour may be a necessary component of a holistic approach to sustainable food policy. Necessary to achieving this aim is an understanding of the determinants of dietary behaviour and how to change consumer behaviour related to lower environmental impact and potentially healthier food choices.

## 2. Livestock and Environmental Impact

According to a report of the Agricultural Organization of the United Nations, production of livestock accounts for 30% of land use globally, and 70% of all agricultural land and is a major contributor to environmental problems related to climate change, freshwater pollution and availability, and biodiversity [5]. The livestock industry is a major contributor to global warming, emitting 18 percent of total greenhouse gas emissions, which is a higher share than transport [5]. Of these emissions, the livestock sector contributes 65% of anthropogenic nitrous oxide emissions (mainly from manure), 37% of anthropogenic methane (mainly from enteric fermentation and manure), 9% of anthropogenic carbon dioxide emissions (mainly from land use changes including deforestation), and 64% of anthropogenic ammonia emissions [5]. Given the reduced greenhouse gas emissions if following a plant-based diet relative to an animal-based diet, it has been contended that personal choice of diet is just as important as personal choice about transport [8].

Globally, there are increasing problems of freshwater shortage, with a projected 64% of the population living in water-stressed areas by the year 2025 [5]. The livestock sector is a major contributor to water shortage, being directly responsible for over 8% of global human water use [5]. In Australia the dairy industry is the highest user of irrigated water in the Murray-Darling Basin [9]. Livestock also negatively impact on the replenishment of freshwater, through compacting soil (thereby reducing infiltration), contributing to deforestation (thereby increasing runoff), degrading the banks of watercourses, lowering water tables, and reducing dry season flows [5].

Biodiversity is rapidly decreasing with the loss of species estimated between 50 to 500 times greater than prior rates found in fossil records [5]. Livestock's threat to biodiversity is largely through occupying land that was once a habitat for wildlife. The highest rate of deforestation is currently occurring in Latin America, where 70% of previously forested land in the Amazon is now used for livestock pastures [5].

Pimentel and Pimentel [10] contend that the current human dietary pattern evident in North America is unsustainable with respect to environmental impact. Based on their own research and statistics from the Department of Agriculture, they stated that producing the equivalent amount of protein from meat takes 11 times the amount of fossil fuel use compared to a vegetable-based protein with corn being the example used for the calculations. Further, producing the equivalent amount of animal protein takes 100 times more water than for vegetable protein. Much of this use comes from growing the crops and forage for livestock with agricultural irrigation accounting for 85% of freshwater use. It was concluded that current animal-based diets and population growth threaten sustainable use of natural resources [10].

Reijnders and Soret also questioned the sustainability of agriculture as practiced today [11]. The authors reviewed recent European studies to determine the impact of dietary choices on the environment and found large disparities in the amount of resources required to produce different foods and their impact on the environment. In regards to meat and soy products, an equivalent amount of meat protein requires 6 to 17 times the amount of land than soy protein. Also, meat production requires 4.4 times the amount of water through intensive irrigation, and 26 times the amount of water through rainfall alone, compared to soy. Meat production also requires between 2.5 and 50 times (depending on the intensity of agriculture) more use of fossil fuels than soy protein requires.

Baroni et al. conducted a comprehensive assessment of the impact of different dietary patterns [12]. The diets they examined were classified into seven types with normal classifying meat consumption: “normal” with conventional farming; “normal” with organic farming; vegetarian with conventional; vegetarian with organic; vegan with conventional; vegan with organic; and “normal” Italian with conventional farming. A registered dietician developed a seven-day diet plan for each dietary pattern. The impact of each dietary plan was assessed using the Life Cycle Assessment method. This necessitated assessing the whole stage, “from the extraction and processing of raw materials to the production, transportation, distribution, use, reuse, recycling, and final disposal” [12, page 2].

From this analysis it was discovered that the vegan diet had the lowest impact on the environment [12]. Organic farming was also better for the environment than conventional methods. In assessing the impact of single food items, beef had the largest impact, followed by fish, cheese, and milk. The sources of stress were from waste produced that could not be used as fertiliser, land use, fossil fuel use and water use. The use of water for irrigating lands and crops to feed cattle was noted as an inefficient use of natural resources and unsustainable to feed future generations. It was also noted that land clearing in developing countries is often used for grazing and crop feeds for animals consumed in western countries rather than the crops being used to feed local populations [12]. Similar research has also found that nonvegetarian diets have a higher environmental cost with respect to water use, energy consumption, and fertilizer and pesticide use [13] with beef having a particularly high environmental impact [13, 14].

McMichael et al. investigated ways to reduce the impact of livestock production on the environment [2]. Improved environmental practices, such as improved practices in relation to reducing methane production from livestock enteric formation and manure management [15] were cited as recommendations, however current efficiency measures, were noted as not producing the amount of change required to significantly impact on emissions. Thus it was proposed that western countries significantly reduce their red meat consumption and that developing countries aim to reach this lower target. Labeled as a constriction and convergence policy, this was argued to be the most equitable way of addressing the problem. Increased communication, pricing signals, and policies which reduce population growth were all recommended to address the significant problem of ensuring that resources are able to meet future population needs [2]. The authors concluded that there are clear environmental benefits of plant-based diets.

The evidence suggests wide-scale adoption of a plant-based diet would reduce human impact on the environment and improve some of our most serious environmental problems such as climate change and fresh water scarcity. In addition, a plant-based diet is a more socially equitable means of feeding the earth's population [2]. Any reduction in meat consumption made for environmental reasons may also improve public health profiles around chronic diseases of cardiovascular disease [16], type 2 diabetes [17], and colorectal cancer [18]. It may also result in a decreased threat of zoonotic disease [2].

## 3. Plant-Based Diets and Human Health

Research on health outcomes of a vegetarian diet has shifted from 20 to 30 years ago from questioning the nutritional adequacy of a plant-based diet to that of its potential in preventing and treating disease [19, 20]. People who exclude all animal-based products are recommended to take B_12_ supplements or fortified foods [19]. Vegetarian diets can also be low in omega 3 fatty acids and it is recommended that vegetarians look at how to supplement their diets to ensure adequate intake [21]. As long as recommendations like these are followed, it was concluded by the American and Canadian Dietetic Associations that a wholly plant-based diet is appropriate for all life stages [19]. While levels of iron and zinc have been shown to be lower among people consuming a vegetarian diet, there is no research to suggest that this has any adverse health consequences and there are no differences in anemia prevalence rates between vegetarians and nonvegetarians [22].

An analysis of five prospective studies that compared vegetarian participants with nonvegetarian participants similar in lifestyle characteristics found that there was a significant reduction in mortality from ischemic heart disease but no overall difference in total mortality [23]. Overall both groups had significantly lower mortality rates compared to standardized norms [23]. This could be seen in the Oxford vegetarian study where the 6,000 vegetarian participants were asked to recruit a friend or relative of similar lifestyle and social class [24]. There was a reduction in mortality from ischemic heart disease in the vegetarian group, but both groups were significantly less likely to have died compared to standardized population norms [24]. According to the authors the most striking result in the Oxford study was the highly significant increased risk of mortality from ischemic heart disease according to increased consumption of animal fats and total cholesterol.

In a review of the link between reduced meat consumption and increased longevity it was concluded that while studies have found a relationship between reduced meat consumption and increased longevity, methodological problems require that further research is undertaken to determine causal relationships. The difficulty in delineating between the groups on meat consumption is evident when those eating meat less than once a week are considered nonvegetarian for group analysis purposes [25]. Further some studies have used the self-definition of vegetarian to separate participants, which may not reflect eating patterns as many self-described vegetarians eat meat. An American study found that although the overall diets of self-described vegetarians was healthier than nonvegetarians, two-thirds of self-described vegetarians still ate red meat, poultry, or fish based on a two-day diet recall [26]. Some of these methodological flaws reduce the power to detect a difference between vegetarians and nonvegetarians and the relationship between reduced meat consumption and longevity could be stronger than results indicate [25]. It could also be the protective effect of plant-based foods rather than elimination of animal-based food that is the important factor in determining health outcomes [25].

A review of the literature on plant-based diets and cardiovascular disease concluded that rather than concentrating on total fat consumption the type of fats is very important [16]. A diet consisting of whole grains, unsaturated fats from sources like nuts, high consumption of fruits and vegetables, and adequate intake of n-3 fatty acids can positively impact on reducing the risk of CVD [16]. With this type of diet it was concluded that animal products could be included such as fish, poultry, and low-fat dairy [16]. A study of 34,192 Seventh-Day Adventists found that decreased beef consumption among men was related to a decreased rate of ischemic heart disease (IHD) [27]. It was not clear whether this was due to the negative health consequences of beef consumption such as increased saturated fat or the positive factors of an increase in vegetable and fruit consumption among vegetarians. It was concluded that only comparing diet and health outcomes among Seventh-day Adventists should reduce the effect of confounding variables such as smoking and alcohol consumption, which is low among all participants of this group unlike diet, which is more variable. However, such variables cannot be eliminated as possible mediators for these results.

A review of weight and vegetarian status by Berkow and Barnard [28] found 40 studies examining this relationship. Of these, 29 found that people consuming a vegetarian diet had significantly lower BMI or body weight and a further nine found a lower but not significant reduction, which was mainly due to small sample size. There were two studies that found a nonsignificant increase in body weight or BMI among vegetarians, but this was again confounded by small sample sizes and the inclusion of “health-conscious” volunteers. The possible mechanisms for this reduction in body weight are reduced energy intake, higher consumption of fibre and carbohydrates that are less energy dense, and lower intakes of fats that are energy rich. The vegetarian diets, while consuming adequate levels of proteins, are generally lower in protein than nonvegetarians and this may also relate to body weight. It was concluded that the health benefits of vegetarian diets in reducing risk of chronic diseases may be mediated in part by lower body weight [28].

A position paper of the American and Canadian Dietetics Associations commented that vegetarians have been found to have lower rates of prostate and colon cancer and cancer rates overall but separating dietary from other possible reasons for this relationship is difficult [19]. The 2007 report of the World Cancer Research Fund and American Institute for Cancer Research concluded that meat consumption increased the risk of colorectal cancer but did not make the same conclusion for other cancers [18]. The link between breast cancer and meat consumption has produced more conflicting results compared to the more consistent associations between cancers of the gastrointestinal system and meat consumption [29].

It has been recommended that people at risk of cardiovascular disease and cancer adopt vegetarian diets [30]. It has also been proposed as a means to treat type 2 diabetes [17]. An intervention study for type 2 diabetes participants that deliberately did not involve an exercise component to isolate the effects of diet found that those patients on a vegan diet program made significant improvements in biomedical markers for the disease. While both the vegan diet and control group, which followed the recommended American Diabetes Association (AMA) guidelines, showed significant improvement; the changes were larger in the vegan diet group. In addition the drop-out rate was lower in the AMA group and the authors concluded that it may be easier to comply with the vegan diet as the AMA diet contains restrictions on portion sizes which people find difficult to maintain [31].

When considering the health benefits of plant-based diet, this review did not discuss the food-borne diseases that arise from animal based foods [32]. While universal adoption of plant-based diets was not considered a plausible option, diseases of animal origin that are threats to humans, such as bird flu, would cease to exist if animals were not used for food [33]. However, it has been argued that the link between human's use of animals for food and increased risk of zoonotic diseases can be made public and addressed [34].

## 4. Shifting Dietary Practice

While there may be strong environmental and health benefits for reducing meat consumption, it would appear that the public is only focused on the health benefits. In a street intercept survey 60% listed transport options as a way of helping the environment from an unprompted question but only 3% mentioned diet [35]. Similarly when a sample of college students were asked what were the benefits of a plant-based diet, males only provided health reasons and this was also true of female participants apart from 8% of females that mentioned reduced harm to animals as a benefit [36]. Nor do environmental reasons seem to be the focus of people changing to plant-based diets. Qualitative research conducted with vegetarians and vegans recruited through an online forum found that participants reflected that health or ethical reasons were the initial motivations for adopting meat free diets [37, 38]. Those that started for ethical reasons were seen as distinct from those that were motivated for health reasons and some people commented that they were doing this in spite of the negative health consequences related to concerns about nutritional adequacy of their diets [38]. Only one out of the 33 respondents listed environmental benefits as their initial motivator for change [37]. It was noted that while initial motivations may focus on one aspect such as health or ethical issues, once having adopted a vegetarian diet the self-identity develops and motivations broaden to include environmental benefits and a combination of health and ethical concerns [37].

The research conducted to date on plant-based diets has concentrated on the health and ethical dimensions. Lea et al. [39] conducted a questionnaire study on barriers and perceived benefits of consuming a plant-based diet as well as a focus group study of barriers and benefits of plant-based foods [40]. Among many areas mentioned, the focus groups revealed that barriers such as convenience and ease of preparation were important [40]. The main barrier to eating a plant-based diet was lack of information with 42% agreement of needing more information [39] such as on nutrition and preparation of plant-based meals. A lack of interest in changing and concern that other family members would not change were also popularly identified as barriers. Female participants were less likely to agree that humans were meant to eat meat. The major perceived benefits of eating a plant-based diet were health related with 79% agreeing that it would lower cholesterol and 70% agreeing that it would prevent disease in general [39].

A similar study was conducted previously by Lea and Worsley [41] on a similar sized sample of 603 participants in South Australia. This questionnaire asked specifically about the benefits and barriers to adopting a “vegetarian diet” as opposed to a “plant-based diet,” the term used in the survey published in 2006 [39]. The main barriers were enjoying eating meat (78% agreement), not wanting to change eating habits (56%), thinking that humans were meant to eat meat (44%), family consumption of meat (43%) and needing more information about vegetarian diets (42%) [41]. The perceived benefits were health related but not as high in the 2006 study. That the barriers were much higher and benefits lower than in the more recent survey was commented upon by Lea et al. [39] suggesting that switching to a plant-based diet was seen as a smaller shift than adopting a vegetarian diet. Such information is important for those planning public health campaigns aimed at increasing the consumption of plant-based foods.

Wyker and Davison [36] tested the applicability of the Theory of Planned Behavior [42] and the Transtheoretical Model [43] to predict intention to follow a plant-based diet. They suggested that promoting a plant-based diet could potentially be an effective approach to preventing chronic disease [36]. They found that the decision process to eat more fruit and vegetables was distinct from the decision process to adopt a plant-based diet. They also found that attitudes towards a plant-based diet differed according to gender and their particular stage of change. It was concluded that these findings were important for behaviour change campaigns as those already in the contemplation stage would probably not change based on what health experts suggest. However, they may benefit from information on health benefits and how to obtain plant-based protein which was a common concern for males and females [36].

From a different perspective, a qualitative study of adults from Copenhagen investigated people's attitudes towards meat consumption [44]. In depth interviews were conducted with 11 women, 1 man, and four couples. Sponsored in part by a livestock organisation the interest was people's current perception of meat consumption. There was a gender theme with many women indicating a willingness to stop eating meat except that their children needed meat for protein and that their male partners wanted to eat meat. The authors commented that there was a lot of negative reaction to meat consumption such as negative health consequences and concern over animal treatment. However, they suggested that this was probably not reflective of a reduction in consumption but a shift away from meat being considered the central focus of a meal to being one aspect along with vegetables and cereals. While this was surmised as a process happening for everyday meals, meat still formed the central component of festive and weekend meals [44].

There has been considerable research investigating teenage interest and motivation for vegetarian diets [45, 46]. Studies have found that female adolescents who followed some form of vegetarian status are more likely to have tried weight loss strategies [47]. Baines et al. [48] discovered that of a sample of 9,689 young females aged 22–27 drawn from the Australian Longitudinal Study on Women's Health (ALSWH), those that reported excluding all meat (vegetarians) and those restricting red meat only (semivegetarians) had lower BMI and increased physical activity. However they were much more likely to have poorer mental health as measured by the SF-36, increased likelihood of having been diagnosed with a mental health problem, low iron, reported self-harm, reported menstrual problems, and more likely to be taking medications for depression [48]. Semivegetarians were also more likely to report asthma, tiredness, and skin problems compared to both nonvegetarians and vegetarians [48].

Baines et al. [48] acknowledged that the relationships do not signify causation and that the higher rate of constipation among the vegetarians and semivegetarians may reflect a higher rate of eating disorders among the sample restricting meat-based food. This suggestion was based on previous studies that have found higher rates of eating disorder behaviours among adolescent vegetarians [49]. Major findings of the study of increased reporting of depressive problems and menstrual problems are also features of eating disorders [50]. The study of 4,746 adolescents from Minnesota found that those describing themselves as vegetarian were far more likely to have been told by a doctor they had an eating disorder, weigh themselves frequently, be dissatisfied with their bodies, and practice unhealthy weight control practices [49]. In this study the vegetarians were less likely than semivegetarians to be engaged in eating-disorder-type behaviour and were considered to be healthier and more “stable” than semivegetarians. Their motivations for becoming vegetarian also differed with vegetarians reporting more concern about animal welfare and it was surmised that the semivegetarians were practicing red meat constriction as another form of weight control [49].

Poor body image and eating habits are significant public health concerns in Australia and many other developed countries [51]. The WA Child Health Survey found that only 42% of adolescent females aged 12 to 16 years reported that they felt their weight was about right. Over half (53%) of all adolescent females reported trying to lose weight as compared with 18% of males [52]. Poor eating habits and dieting are closely linked with the development of eating disorders [53]. In a thoughtful paper, O'Dea [54] raises a number of issues when well-meaning programs can have adverse effects by commenting how obesity prevention programs can encourage the uptake of eating disorder behaviours among adolescents. Messages that focus on overweight and obesity are likely to make young people feel worse about their bodies and themselves.

Any promotion of adopting a plant-based diet needs to carefully consider how young people respond to the message and what behavioural effect this may produce. While increasing the consumption of plant-based foods is associated with health benefits, adoption of such a diet during adolescence needs to be carefully managed and can be considered both a risk of weight control practices that may require professional support and an opportunity to assist adolescents to practice a healthy vegetarian diet [49].

## 5. Conclusion

Clearly more research is required on how to promote plant-based diets from a health perspective [36] and environmental perspective. Early findings would suggest that consumers will respond more readily to a campaign emphasising health rather than environmental benefits and for some people an ethical aspect might influence dietary behavior. Motivations may vary according to stages of change, age, and gender [36]. Behavioural change principles were used effectively in a successful mass media promotion campaign to increase fruit and vegetable consumption in Western Australia [55]. Formative research undertaken in this study discovered that people realised the benefit of fruit and vegetable consumption, but there were barriers related to incorrect perceptions of recommended serving size, and lack of time and knowledge about how to prepare meals with vegetables. Objectives and marketing messages directly targeted these barriers and population health surveys found there was an increased consumption of fruit and vegetables over time [55]. Thus there is evidence that well-developed messages based on a thorough understanding of the determinants of that behavior can influence dietary practice.

There are reservations though, about social marketing campaigns and health promotion approaches that focus only on changing behavior without consideration of the broader social and environmental factors influencing that behavior [56]. To ensure sustainable change, any attempt to influence dietary behavior would need to occur within a broader food and social policy agenda. Health promotion has produced a number of successes most noticeably in the areas of reducing smoking rates, decreasing road fatalities, and decreasing rates of cardiovascular disease [57]. The strength lies in a multidisciplinary approach incorporating economic, organisational, policy, and education interventions [58]. Rarely have educational interventions on their own yielded behavioural change but can support and augment other interventions [59]. Thus while increasing education may not directly alter behavior, it may increase attitudes and knowledge and lead to increased support for economic, organizational, and policy interventions that might be more effective in driving change.

Further research is required on what messages might resonate to support broader food policy changes and the medium through which these messages are delivered. One option may lie with the movement in participatory media and social networking, which has enabled public citizens, particularly young people, to be media makers in contrast to the more passive role of media consumers [60]. It has been recommended that young people are skilled in how to use these different media forms effectively to engage as citizens [60]. Equally this opportunity to voice and shape opinion is available to advocacy groups and environmental and public health organisations with an interest in promoting sustainable food choices. Further research is required on how to increase their capacity to utilise these media forms effectively for engaging the public based on a sound understanding of health and social communication principles and evidence. From this research base, environmental and public health agencies could be leading community education and debate on the benefits of a plant-based diet as an important contribution to improving public health and within a broad strategic approach in moving towards a sustainable food system.
